# Exogenous pulmonary surfactant prevents the development of intra‐abdominal adhesions in rats

**DOI:** 10.1111/jcmm.12758

**Published:** 2016-02-01

**Authors:** Alberto Schanaider, Ricardo Cotta‐Pereira, Paulo C. Silva, Hugo Macedo‐Ramos, Johnatas D. Silva, Pedro A. C. Teixeira, Vera L. N. Pannain, Patricia R. M. Rocco, Wagner Baetas‐da‐Cruz

**Affiliations:** ^1^ Postgraduate Program in Surgical Science Department of Surgery School of Medicine Federal University of Rio de Janeiro Rio de Janeiro Brazil; ^2^ Centre for Experimental Surgery Department of Surgery School of Medicine Federal University of Rio de Janeiro Rio de Janeiro Brazil; ^3^ Translational Laboratory in Molecular Physiology Centre for Experimental Surgery Department of Surgery School of Medicine Federal University of Rio de Janeiro Rio de Janeiro Brazil; ^4^ Postgraduate Program in Biological Sciences – Physiology Carlos Chagas Filho Institute of Biophysics Federal University of Rio de Janeiro Rio de Janeiro Brazil; ^5^ Laboratory of Pulmonary Investigation Carlos Chagas Filho Institute of Biophysics Federal University of Rio de Janeiro Rio de Janeiro Brazil; ^6^ Laboratory of Glycobiology Carlos Chagas Filho Institute of Biophysics Federal University of Rio de Janeiro Rio de Janeiro Brazil; ^7^ Department of Pathology School of Medicine Federal University of Rio de Janeiro Rio de Janeiro Brazil

**Keywords:** peritoneal adhesions, gastrointestinal surgery, inflammation, matrix metalloproteinase, transforming growth factor‐β, type III procollagen

## Abstract

Intra‐abdominal adhesions are major post‐operative complications for which no effective means of prevention is available. We aimed to evaluate the efficacy of exogenous pulmonary surfactant administration in the prevention of post‐operative abdominal adhesions. Rats were randomly assigned to undergo laparotomy (L) or gastroenterostomy (GE) and then treated with surfactant (groups L‐S and GE‐S, respectively). Intra‐abdominal adhesions, collagen fibre content, metalloproteinase (MMP)‐9, expression of growth factors (TGF‐β, KGF and VEGF), type III procollagen (PCIII) and pro‐caspase 3, as well as isolectin B4 and ED1‐positive cells expressing MMP‐9, were evaluated. Groups treated with surfactant (GE‐S and L‐S) exhibited fewer adhesions. A significant reduction in collagen fibre content was observed in GE‐S compared to GE animals (*P* < 0.001). *In situ* and gelatin zymography analysis showed higher MMP‐9 expression and activity in the GE‐S group compared to the GE group (*P* < 0.05). ED1‐positive cell counts were significantly higher in the GE‐S group (*P* < 0.001) than in the GE group. Virtually all cells positive for ED1 were MMP‐9+. Double‐labelling of MMP‐9 with IB4 showed no significant differences between GE‐S and GE groups. TGF‐β, KGF, PCIII and pro‐caspase‐3 mRNA expression decreased significantly in GE‐S compared to GE animals (*P* < 0.05). Surfactant administration also reduced apoptosis in the GE‐S group. These findings suggest that surfactant reduces the intra‐abdominal adhesions triggered by laparotomy and gastrointestinal anastomosis, thus preventing fibrosis formation at the peritoneal surfaces. This preclinical study suggests an innovative treatment strategy for intra‐abdominal adhesions with surfactant and to endorse its putative mechanism of action.

## Introduction

Intra‐abdominal adhesions consist of fibrotic bands between organs and tissues formed as a result of injuries to the peritoneal cavity (surgical intervention, blunt and penetrating trauma, foreign bodies), as well as of infections and inflammatory processes 1. Intra‐abdominal adhesions can cause several complications, even years after a peritoneal injury 2. The most frequent consequence of this disorder is mechanical sub‐occlusion or obstruction of the bowel, and the most life‐threatening consequence is bowel strangulation. Treatment of these complications can place a high social and financial burden on the health services because of recurrent surgical interventions 3, 4. Although the pathophysiology of intra‐abdominal adhesions has been extensively investigated, there is currently no drug or therapeutic strategy able to prevent it in an effective manner 2.

The formation of intra‐abdominal adhesions is closely related to the mesothelial cells of the visceral and parietal peritoneum. Any injury can lead to exposure of collagen and von Willebrand factor, both present in the sub‐endothelial layer, thus causing platelet activation 5. In turn, platelet activation and chemotaxis of monocytes will promote the release of transforming growth factor (TGF)‐β by macrophages, stimulating the synthesis of extracellular matrix by local fibroblasts and subsequent progression of fibrosis 6. In addition to its role as a pro‐fibrotic cytokine, TGF‐β can directly induce the differentiation of fibroblasts into collagen‐secreting myofibroblasts, which facilitates fibrogenesis 7. Metalloproteinases (MMPs) play important roles in regulation of the tissue microenvironment, mediating remodelling through cell–cell and cell–matrix contact in both physiological and pathological processes, even during anastomotic healing 8.

Recently, some authors reported prevention of adhesions and improvement of histologic findings by administration of two different pulmonary surfactants (Poractant and Beractant) in a rat model of caecal injury after abrasion and topical administration of alcohol 9.

Several other therapeutic approaches have also been suggested for the prevention of intra‐abdominal adhesions, but none has successfully reached the clinical trial stage 10.

This study sought to test the effects of exogenous pulmonary surfactant on the prevention of intra‐abdominal adhesions and to better characterize the mechanisms of action of surfactant in this setting.

## Material and methods

### Animals and experimental design

Male Wistar rats (*Rattus norvegicus*), specific‐pathogen‐free, with body weight ranging from 250 to 300 g, were obtained from the Centre for Experimental Surgery Laboratory at the Federal University of Rio de Janeiro, Brazil. All protocols were reviewed and approved by the Institutional Animal Care and Use Committee of the Federal University of Rio de Janeiro (no. 84/09/2). Thirty‐six rats were randomly assigned across four groups of nine animals each. Group L was subjected to laparotomy alone. Group LS rats were subjected to laparotomy and treated with surfactant. Group GE rats were subjected to gastroenterostomy. Group GE‐S rats were subjected to gastroenterostomy and treated with surfactant.

### Gastroenterostomy for induction of intra‐abdominal adhesions

Animals were anaesthetized with a combination of ketamine (100 mg/kg) and xylazine (10 mg/kg), administered intraperitoneally. An anterior midline incision was made on the upper third of the abdominal wall, and a gastroenterostomy was performed with a 6–0 polypropylene suture. The post‐operative care protocol included early oral feeding with a fluid diet, beginning approximately 12 hrs after the gastroenterostomy, which was altered to a solid diet on the third day. The rats were euthanized 28 days later under general anaesthesia. A U‐shaped incision was made to allow immediate inspection of adhesions, which were evaluated according to a standard scoring system adapted from Zühlke *et al*. 11.

### Surfactant

Immediately after the surgical procedure, each animal was treated with topical application of 2 ml of Beractant (Survanta^®^, Abbott Park, IL, USA), dripped over the gastric area (group L‐S) or the gastroenterostomy area (group GE‐S). Beractant is a natural sterile and non‐pyrogenic bovine lung extract containing 25 mg/ml phospholipids (including 11.0–15.5 mg/ml disaturated phosphatidylcholine), 0.5–1.75 mg/ml triglycerides, 1.4–3.5 mg/ml free fatty acids and less than 1.0 mg/ml protein (two hydrophobic, low‐molecular weight, surfactant‐associated proteins commonly known as SP‐B and SP‐C), suspended in 0.9% sodium chloride solution (saline solution).

### Histology

In all experiments, the left middle and lateral liver lobes and the anastomotic area containing the bowel loop were excised en bloc, fixed in 10% neutral‐buffered formalin and processed into paraffin sections for staining with haematoxylin and eosin (Vector Laboratories, Burlingame, CA, USA). Total collagen content was evaluated using a Picrosirius Red (PSR)/Fast collagen detection kit (Chondrex Inc., Redmond, WA, USA). For histomorphometry, we used an image analysis system composed of a light microscope (Eclipse E800; Nikon, Tokyo, Japan) coupled to a digital camera (Evolution Media Cybernetics Inc., Bethesda, MD, USA) and a computer running QCapture 2.95.0 software, version 2.0.5 (Silicon Graphics Inc., Milpitas, CA, USA). After adjustment of software settings and calibration, high‐resolution images (2048 × 1536 pixel buffer) were captured using a 40× objective lens. To measure collagen content, 20 photomicrographs of gastroenterostomy sites were obtained, separately, from sections stained with Sirius Red. The results are expressed as percentage of reactive tissue in the total area.

### Gelatin zymography

Gelatin zymography was performed as described elsewhere 12. The expression and activity of MMP‐9 were investigated in all rats of groups L, LS, GE and GE‐S by SDS‐PAGE gelatin zymography using a 4% polyacrylamide stacking gel and a 10% polyacrylamide resolving gel (Bio‐Rad Co., Richmond, VA, USA) containing 1.5 mg/ml gelatin (BDH Laboratory Supplies, Poole, UK). Equal amounts of prepared protein samples (20 μg) were mixed with SDS sample buffer under non‐reducing conditions. After electrophoretic separation, the gel was renatured with 2.5% Triton X‐100 for 30 min. and then incubated with developing buffer (50 mM Tris–HCl pH 8.0, 2.5 mM CaCl_2_) at 37°C overnight. After developing, the gel was stained with 0.25% Coomassie Blue R‐250 for 45 min. and then destained appropriately. Proteolytic bands in the zymography were quantified by scanning densitometry using the ImageJ 1.46 program (United States National Institutes of Health).

### *In situ* zymography

For localization of gelatinolytic activity, *in situ* zymography was performed as described by Mira *et al*. 13. Metalloproteinase activity was measured in frozen samples from the L, LS, GE and GE‐S using DQ Gelatin (E12055; Molecular Probes, Eugene, OR, USA) as a fluorogenic substrate. These samples were embedded in Tissue Tek and cut into 5‐μm sections with a cryostat (Leica, Buffalo Grove, IL, USA). Sample sections were incubated with 1.0 mg/ml DQ gelatin in Tris‐CaCl_2_ buffer (50 mM Tris, 10 mM CaCl_2_, 1 mM ZnCl_2_) in dark humidified chambers for 1 hr. The sections were examined with an Olympus AX70 fluorescence microscope (Olympus America Inc., Center Valley, PA, USA), and images were captured at 400× magnification. Proteolytic activity was detected by a gelatinase/collagenase assay kit (EnzChek; Molecular Probes) as bright green fluorescence, which indicates substrate breakdown, and was evaluated using the ImageJ program (United States National Institutes of Health).

### Histological procedures and double‐labelling

The specimens were briefly embedded in OCT compound and quickly frozen in liquid nitrogen‐cooled isopentane. Sections were cut at a thickness of 5 μm with a Leica cryostat and washed with wash buffer (PBS plus 0.2% Triton X‐100). Then, the sections were incubated overnight at 4°C with primary antibodies diluted with blocking buffer (PBS plus 0.2% Triton X‐100 and 20% heat‐inactivated goat serum). The primary antibodies used were polyclonal anti‐MMP9 (Ad1/20A6; Vector Laboratories) at 1:100. After several washes with buffer, sections were incubated for 1 hr at room temperature with secondary antibodies diluted with blocking buffer. The secondary antibodies used at 1:500 were anti‐mouse Cy‐3 (Sigma‐Aldrich, St. Louis, MO, USA) or anti‐rabbit Alexa488 (Molecular Probes). The sections were counterstained with 4′,6‐diamidino‐2‐phenylindole to detect nuclei, washed several times with wash buffer and mounted in Vectorshield (Vector Laboratories). Metalloproteinase‐9 expression was detected as bright red fluorescence and was evaluated using ImageJ software.

A histochemical assay with biotinylated isolectin B4 (IB4) 1:200 and fluorescein isothiocyanate‐conjugated streptavidin 1:1000 binding was performed to verify the presence of macrophages. To evaluate the presence of ED1‐containing cells (macrophage infiltration), tissue sections were incubated with mouse anti‐ED1 (monoclonal antibody, cat # sc‐59103; Santa Cruz Biotechnology, Santa Cruz, CA, USA) at a 1:1000 dilution in 0.05 M Tris‐HCl buffer containing 2% bovine serum albumin at 4°C overnight. Afterwards, the samples were incubated for 1 hr at room temperature with Alexa flour 488‐goat anti‐mouse secondary antibody (1:200 dilution; Molecular Probes) diluted with blocking buffer. Red fluorescence was visualized by adding a Cy‐3‐conjugated anti‐mouse secondary antibody (1:200, AP160P; Chemicon São Paulo, Brazil) for 1 hr. To confirm antibody specificity, the primary antibody was omitted and substituted with PBS 1% bovine serum albumin. Cy‐3 did not bind nonspecifically to the tissue sections.

### Quantitative RT‐PCR

For quantitative RT‐PCR, central slices of the left middle and lateral liver lobes containing the adhered bowel loop were cut, collected in cryotubes, quick‐frozen by immersion in liquid nitrogen and stored at −80°C. Total RNA was extracted from the frozen tissues using Trizol reagent (Invitrogen, Carlsbad, CA, USA) according to the manufacturer's recommendations. RNA concentration was measured by spectrophotometry in a Nanodrop^®^ ND‐1000 system. First‐strand cDNA was synthesized from total RNA using an M‐MLV Reverse Transcriptase Kit (Invitrogen). PCR primers for target genes were purchased (Invitrogen). The following primers were used: TGF‐β (sense, 3′‐GCCCTGTATTCCGTCTCCT‐5′; antisense, 5′‐ATACGCCTGAGTGGCTGTC‐3′), vascular endothelial growth fator – VEGF (sense, 5′‐CAGAAAGCCATGAAGTGGT3′; antisense, 5′‐ACACAGGACGGCTGAAGAT‐3′), type III procollagen – PCIII (sense, 5′‐CTGCCATTGCTGGAGTTG‐3′; antisense, 5′‐GCAGCCATCCTCTAGAAC‐3′), caspase‐3 (sense, 5′‐GGCCGACTTCCTGTATGC‐3′; antisense, 5′‐GCGCAAAGTGACTGGATG‐3′), keratinocyte growth factor – KGF (sense, 5′‐GTAGCGATCAACTCAAGGTC‐3′, antisense, 5′‐ATTTAAGGCCACGAACATTT‐3′), and glyceraldehyde‐3‐phosphate dehydrogenase (GAPDH; sense, 5′‐GTCTTCACCACCATGGAG‐3′; antisense, 5′‐CGATGCCAAAGTTGTCATG‐3′). Relative mRNA levels were measured with a SYBR green detection system using ABI 7500 Real‐Time PCR (Applied Biosystems, Foster City, CA, USA). Quantitative real‐time RT‐PCR was performed to measure the relative levels of expression of TGF‐β, VEGF, KGF, caspase‐3 and PCIII genes in the L, LS, GE and GE‐S groups. Each gene was studied in triplicate for each animal. The entire set of experiments was performed four times, one for each animal in each group. For each sample, the expression of each gene was normalized to the expression of the housekeeping gene GAPDH using the 2^−ΔΔCt^ method, where ΔCt = Ct, reference gene; Ct, target gene.

### Immunoblotting

Standard Western blotting techniques were used to detect activated caspase‐3. The specimens were removed and homogenized in a lysis buffer containing a cocktail of proteinase and phosphatase inhibitors. The protein concentrations were determined by a bicinchoninic acid protein assay (Pierce, Rockford, IL, USA), and 30 μg of proteins was loaded and separated on SDS‐PAGE gels (4–15%; Bio‐Rad Co.). After transfer, blots were incubated overnight at 4°C with a polyclonal antibody against murine‐activated caspase‐3 (1:1000 dilution; PharMingen, San Diego, CA, USA). Protein bands were visualized using peroxidase‐conjugated secondary antibodies (adsorbed to rat serum proteins; Jackson ImmunoResearch, West Grove, PA, USA) and enhanced chemiluminescence with Hyperfilm (Amersham, Little Chalfont, UK). Quantitation of bands corresponding to changes in protein levels was performed with laser‐scanned densitometric analysis.

### Macrophage–myofibroblast cocultures

Myofibroblast differentiation was induced in fibroblasts by plating at low density according to the method described by Masur *et al*. 14. Peritoneal macrophages were obtained from adult Wistar rats by peritoneal lavage 4 days after intraperitoneal injection of 4% thioglycolate (3 ml). Cells were cultured in 1:1 (vol/vol) DMEM/Ham's F‐12 medium containing 10% (vol/vol) foetal bovine serum (FBS) and antibiotics (100 U penicillin, 100 μg streptomycin, 0.25 μg amphotericin B and 50 μg gentamicin/ml). For phenotypic identification of myofibroblasts, the cultures were incubated with a rabbit polyclonal anti‐alpha smooth muscle actin (anti‐α‐SMA—1:100; Sigma‐Aldrich) and mouse monoclonal anti‐desmin antibody (anti‐Desmin—1:100; Sigma‐Aldrich). After reaction with the primary antibodies of interest, cells were incubated with goat anti‐rabbit IgG and sheep anti‐mouse IgG secondary antibody, labelled either with Alexa 488 or Cy‐3, washed in pH 7.4 PBS, mounted with *n*‐propylgallate PBS‐glycerol and coverslipped. Peritoneal macrophages were obtained from adult Wistar rats by peritoneal lavage 4 days after intraperitoneal injection of 4% thioglycolate (3 ml). Direct coculture was performed by directly adding peritoneal macrophages (1 × 10^6^ viable cells; trypan blue exclusion) to six‐well plates containing a monolayer of differentiated myofibroblast (1 × 10^6^ cells). Then, non‐adherent cells were removed by washing, and the adherent cells were cultured in the same medium supplemented with 10% FBS. Cell culture was followed sequentially with light microscopy at 37°C for up to 72 hrs, with fixation of the cells at 24, 48 and 72 hrs of interaction.

### Statistical analysis

Significant differences among the experimental groups were evaluated using anova followed by Bonferroni's multiple comparison test. The Kruskal–Wallis test followed by Dunn's test was also used. Statistical analyses were performed in GraphPad Prism^®^ software (GraphPad Software, Inc., La Jolla, CA, USA). Results with a *P* < 0.05 were considered significant.

## Results

### Surfactant administration prevented fibrotic band formation *in vivo*

**Figure 1:**
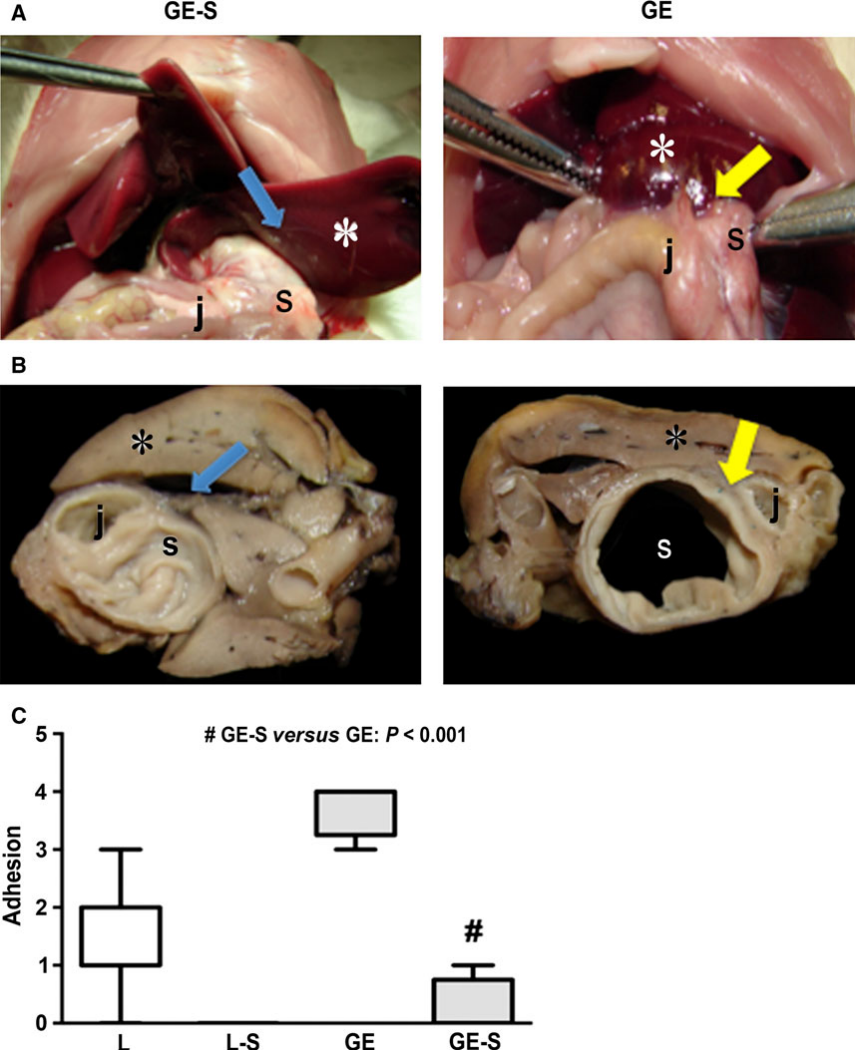
Laparotomy view showing cross‐sections of fresh liver (*), stomach (s) and jejunum (j) samples from GE‐S (left) and GS (right) animals, 4 weeks after the operation. Note (**A**) the absence of adhesions between the undamaged liver capsule and the gastroenterostomy area in an animal treated with surfactant (GE‐S, left) (blue arrow) *versus* firm adhesions (grade 4) in a non‐treated animal (GE, right) (yellow arrow). Gross views of fixed *en bloc* samples of liver tissue and gastrointestinal area (**B**) of representative animals from the GE‐S (left) and GE (right) groups exhibit the same pattern. Blue arrow: absence of adhesions. Yellow arrow: severe adhesions (grade 4). Surfactant administration prevented formation of fibrotic adhesions *in vivo* (**C**). ^#^ *P* < 0.001 for GE‐S *versus* GE.

Gross analysis using a semi‐quantitative score showed that animals in the L‐S and GE‐S groups (treated with surfactant) had reduced levels of adhesion compared to the L and GE (non‐treated groups), respectively (*P* < 0.05). We found a predominance of no detectable adhesions (grade 0) in the groups that received surfactant, whereas higher scores (3 and 4) were observed in non‐treated animals (Fig. 1A–C).

### Surfactant decreased collagen fibre content

**Figure 2:**
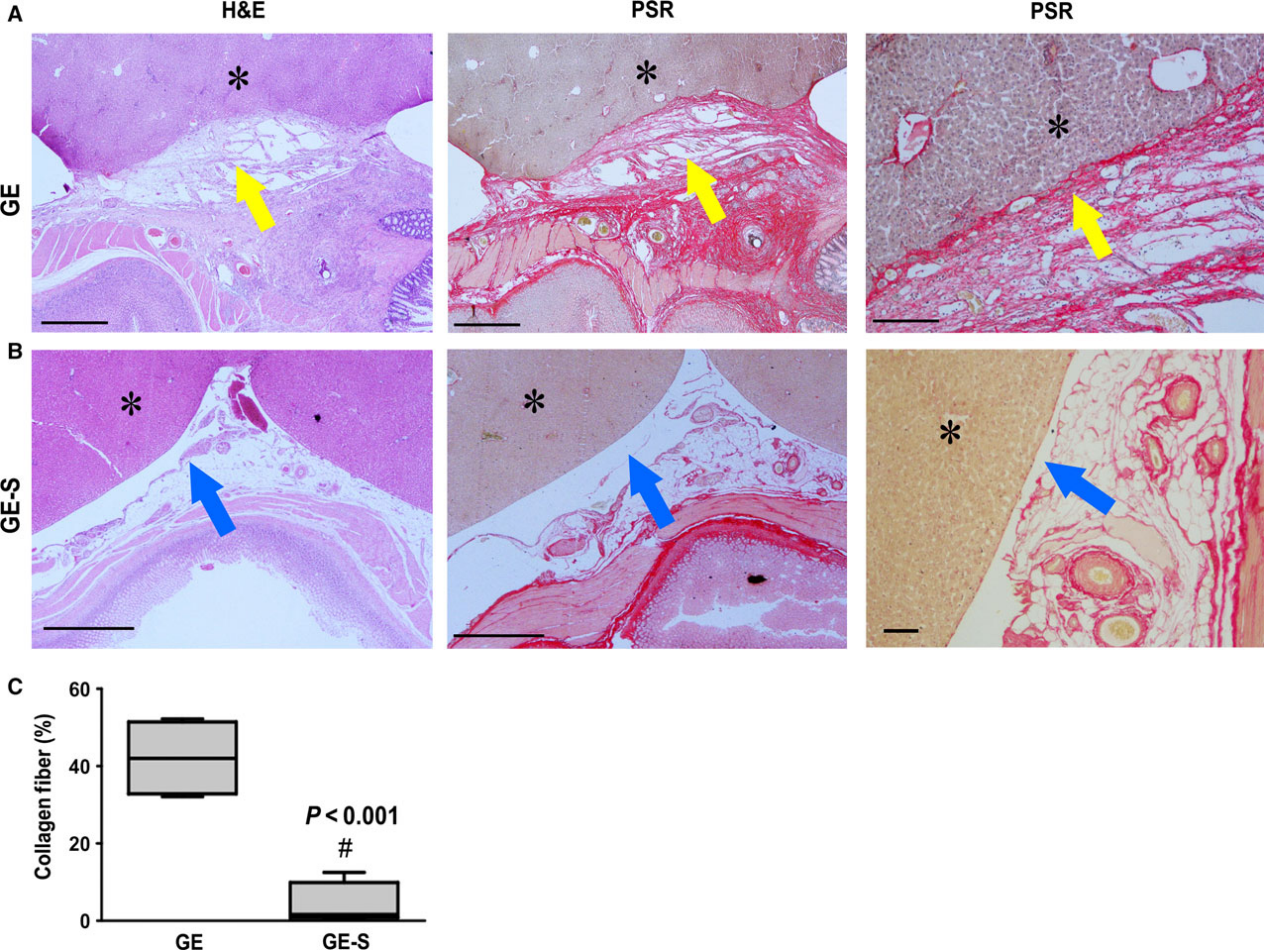
Histopathological examination. Sections obtained from representative GE (**A**) and GE‐S (**B**) animals, stained with haematoxylin and eosin (left) and Picrosirius Red (PSR) (centre, 20× magnification; right, 100× magnification). Note presence of dense acellular tissue (yellow arrow) between the liver surface (*) and the anastomosed region in a GE section (upper row, left). In the same section, PSR revealed intense presence of collagen fibres (yellow arrow) filling the gap between the gastroenterostomy and the liver (upper row, centre and right). Furthermore, fibrous tissue can be seen breaking through the liver capsule (upper row, centre). Implanted hepatocytes are visible amongst the collagen fibres (right). In GE‐S sections (lower row), no fibrous tissue was detectable by haematoxylin and eosin staining (blue arrow). This was confirmed by PSR staining, with negligible presence of collagen fibres (blue arrow) and an intact hepatic capsule, without fibrous tissue contact, scale bar: 1000 μm. Quantitative analysis of collagen fibres showed a significant (*P* < 0.001) reduction in collagen fibre content in the surfactant‐treated group (GE‐S) *versus* untreated controls (GE) (**C**). *# p<0.001*

In the GE group, histopathological examination revealed dense acellular tissue between the liver surface and the anastomosed region in sections stained with haematoxylin and eosin (Fig. 2A). Adjacent sections stained individually revealed a deep red colour consistent with collagen fibres, which became evident after counterstaining with PSR (Fig. 2A). However, these same tests did not show any trace of collagen fibres in animals which underwent gastroenterostomy, and were then treated with surfactant (GE‐S) (Fig. 2B). Only animals with intra‐abdominal adhesions exhibited high amounts of collagen fibre; all other groups exhibited similarly low levels. No significant difference was observed between L and L‐S animals, and a positive, but non‐significant, trend was observed between these groups and the GE‐S group (data not shown). These data were confirmed by quantitative analysis of each group, supporting a significant reduction in collagen fibre content in GE‐S compared to GE animals (Fig. 2C).

### Surfactant administration increased MMP‐9 activity

**Figure 3:**
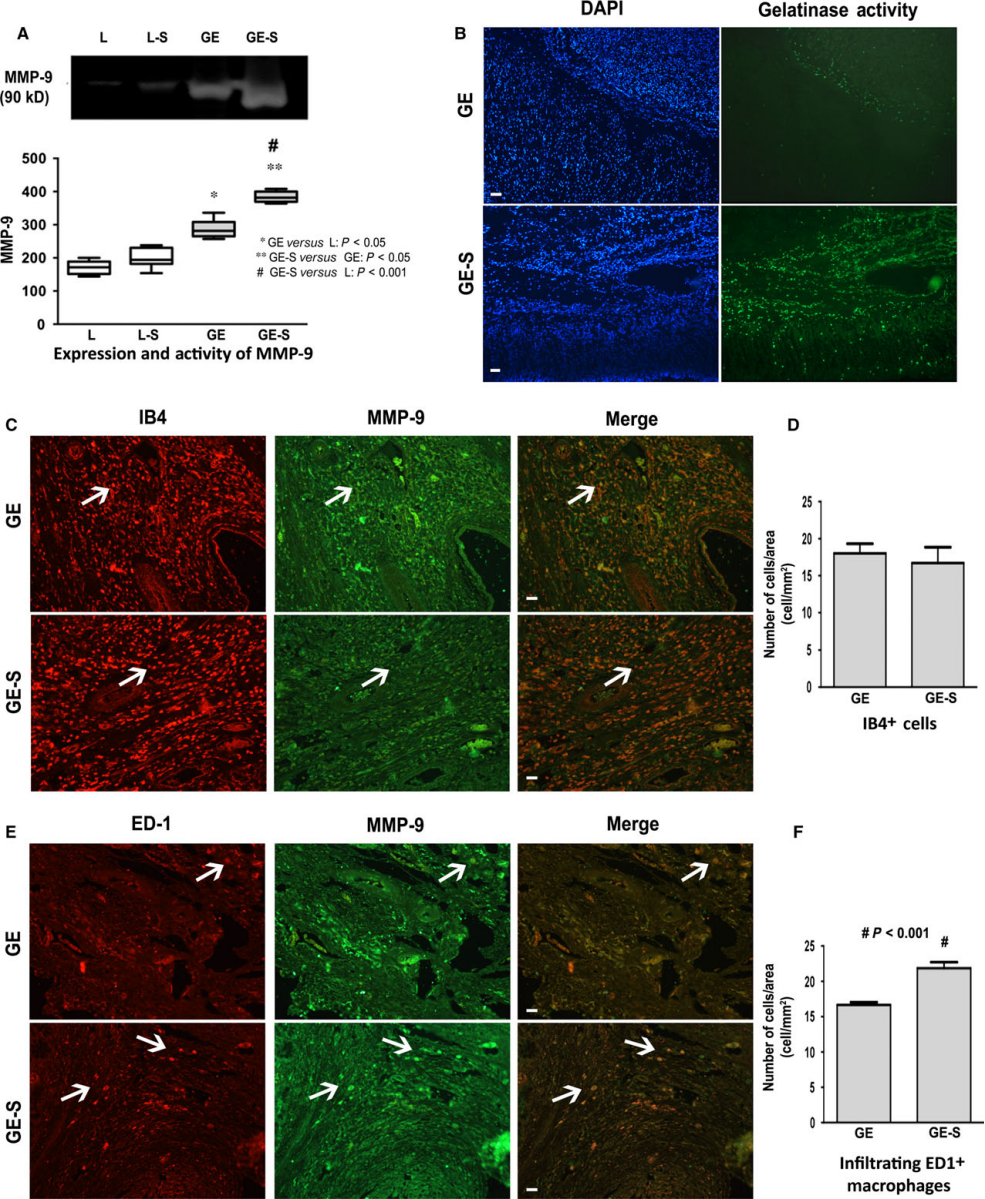
Expression and activity of MMP‐9 *in vivo*. Gel zymography analysis showed that MMP‐9 activity was significantly higher in GE‐S *versus* GE animals (*P* < 0.05) (**A**). *In situ* zymography analysis of MMP‐9 expression showed a larger number of cells positive for gelatinase activity in GE‐S *versus* GE animals. (**B**) Isolectin B4 (IB4) staining of the gastroenterostomy region revealed no significant difference in total number of macrophages (**C**, arrows in the left‐hand column) between the GE and GE‐S animals (**C**, first‐ *versus* second‐row, and **D**). The number of ED1‐positive cells (**E**, arrows in the left‐hand column) was significantly higher (*P* < 0.001) in GE‐S *versus* GE animals (**F**). No significant differences in number of cells double‐labelled for MMP‐9/IB4 were observed between the GE‐S and GE groups (third column in **C**). Note that virtually all cells positive for ED1 were MMP‐9+ (third column in **E**), scale bar: 200 μm.

*In situ* zymography analysis revealed a large amount of cells positive for gelatinase activity in GE‐S compared to GE animals (*P* < 0.05) (Fig. 3B). The same result was observed in gelatin zymography analysis, which showed that MMP‐9 activity was significantly higher in the GE‐S group than in the GE group (Fig. 3A). No significant differences were observed between L and L‐S, and a very low and non‐significant difference was observed between these groups and the GE group (data not shown).

### Surfactant administration increased the number of activated macrophages and tissue levels of MMP‐9

Macrophages were visualized by reaction of the samples with IB4. Double‐labelling of MMP‐9 with IB4 showed no significant differences between GE‐S and GE groups (Fig. 3C and D). However, analysis of the number of activated macrophages immunostained with an anti‐ED1 antibody revealed that the number of ED1+ cells was significantly higher (*P* < 0.001) in the GE‐S group than in the GE group (Fig. 3E and F). In both groups, virtually all cells positive for ED1were MMP‐9+ (Fig. 3E). During counting of ED1 and IB4 double‐positive cells expressing MMP‐9, the total numbers of cells found in groups L and L‐S were very similar. Furthermore, there were no significant differences between these groups and the GE group under the same conditions of analysis (data not shown).

### Surfactant administration decreased mRNA expression of TGF‐B, KGF, PCIII and pro‐caspase‐3

**Figure 4:**
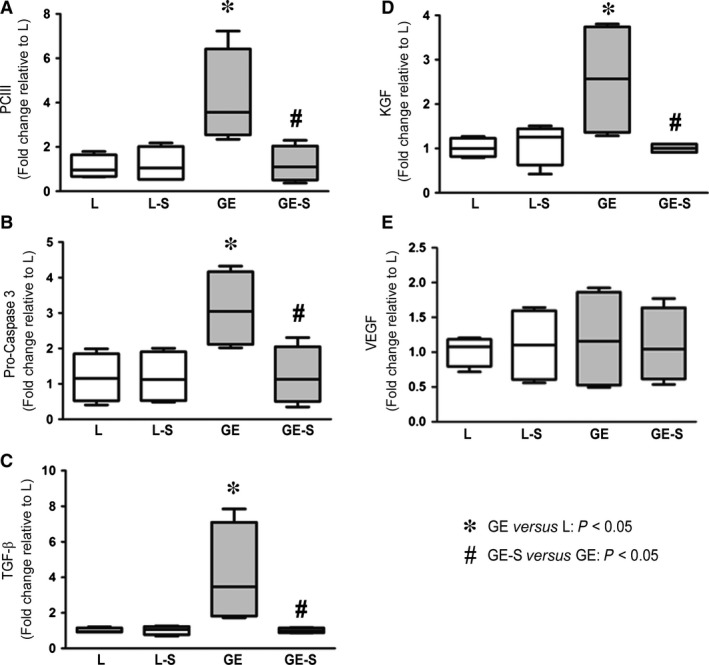
Quantitative RT‐PCR analysis of TGF‐β, KGF, PCIII and pro‐caspase‐3 mRNA. Expression of all genes (**A**–**D**), except for VEGF mRNA (**E**), was significantly reduced in GE‐S *versus* GE animals (*P* < 0.05). Horizontal bars represent medians, boxes represent the 25th and 75th percentiles and vertical bars represent ranges.

Transforming growth factor‐β, KGF, PCIII and pro‐caspase‐3 expression was significantly higher in GE than in L animals, and lower in GE‐S than in GE animals (*P* < 0.05; Fig. 4A–D). No significant difference in VEGF mRNA expression was observed among groups (Fig. 4E).

### Prevention of apoptosis induced by surfactant

**Figure 5:**
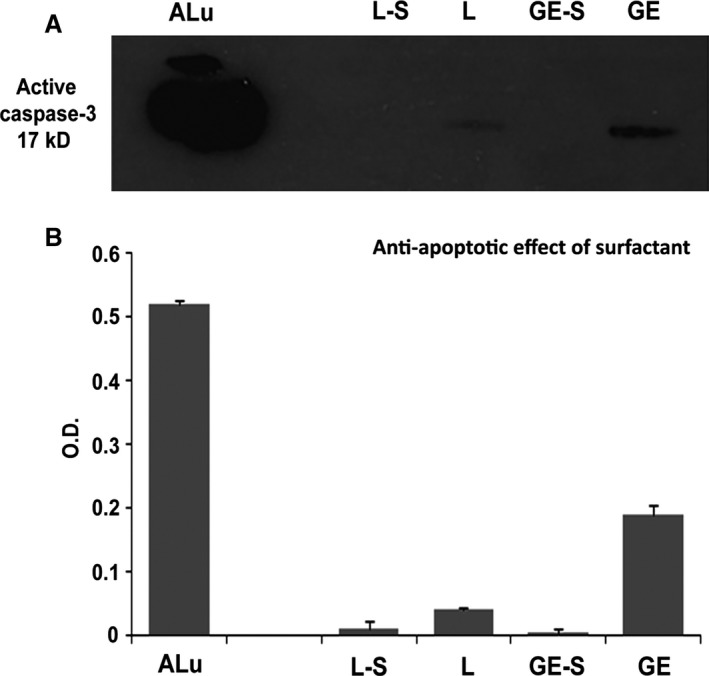
Western blot analysis of activated caspase‐3 expression. Apoptotic lung samples of mice, used as positive controls, showed a strong reaction band (ALu). No band was detected in rats subjected to gastroenterostomy and treated with surfactant (GE‐S). Rats subjected to laparotomy alone (L) showed only a slight reaction, which was not detected after treatment with surfactant (LS). A band corresponding to activated caspase‐3 was observed in rats subjected to gastroenterostomy alone (GE), without surfactant (**A**). Bar graph summarizing densitometric analysis of the bands (**B**).

To test whether apoptosis was present in the gastroenterostomy area of rats treated with or not treated with surfactant, a Western blot analysis of activated caspase‐3 expression was performed. Mouse lung samples were used as a positive control after induction of apoptosis by increased mechanical stress. These samples showed a strong reaction band, confirming the effectiveness of the labelling procedure (Fig. 5–ALu). Rats subjected to laparotomy showed only a slight reaction (Fig. 5—L). However, this band was not detected in rats in the L‐S group (laparotomy plus surfactant, Fig. 5—LS). In rats subjected to gastroenterostomy, a band corresponding to activated caspase‐3 was detected (Fig. 5—GE). However, no band was detected in rats under these same surgery conditions which had been treated with surfactant (Fig. 5—GE‐S). These results suggest that surfactant was able to prevent apoptosis after laparotomy or gastroenterostomy.

### Surfactant administration induced formation of myofibroblast and macrophage clusters *in vitro*

**Figure 6:**
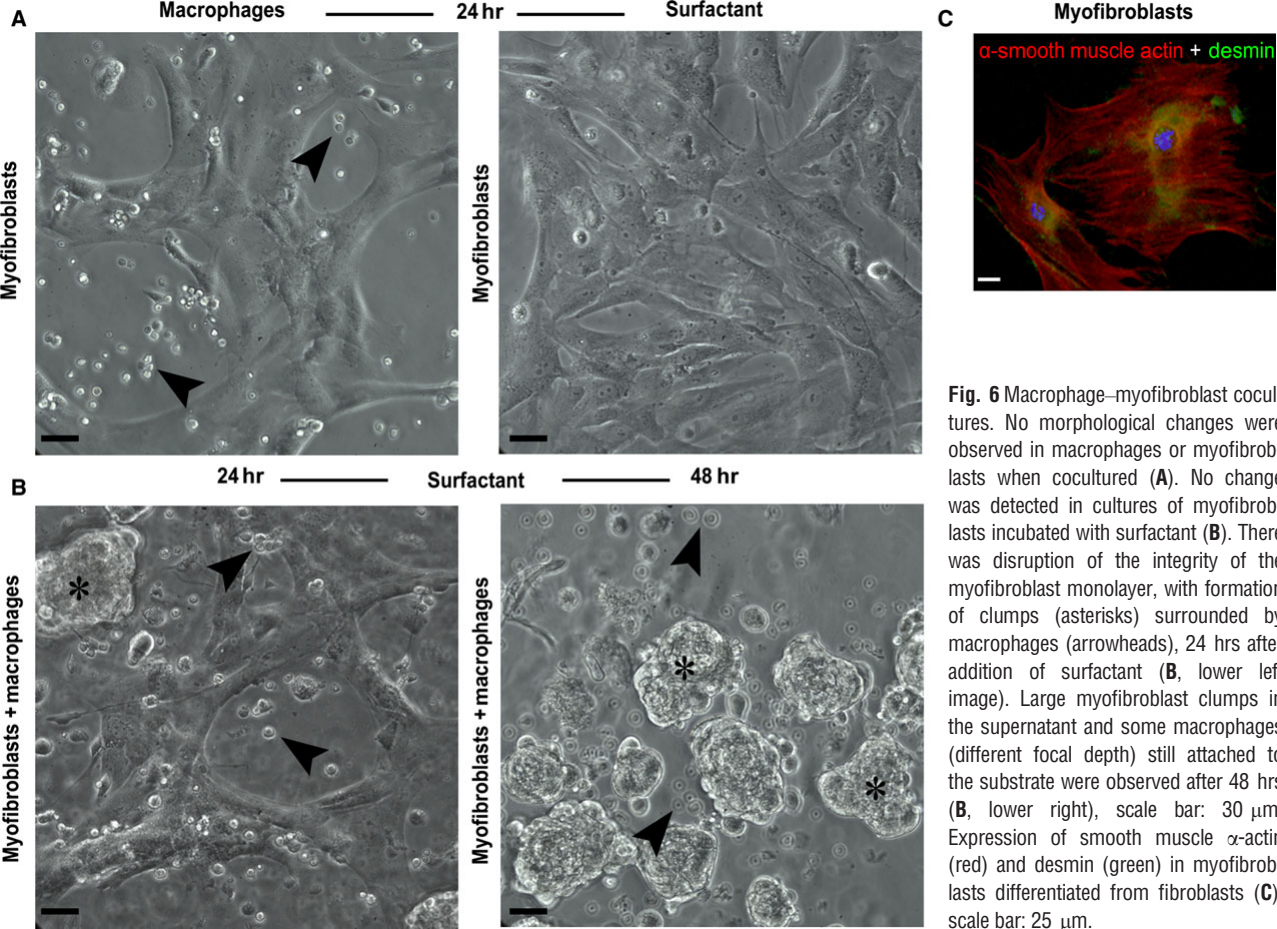
Macrophage–myofibroblast cocultures. No morphological changes were observed in macrophages or myofibroblasts when cocultured (**A**). No change was detected in cultures of myofibroblasts incubated with surfactant (**B**). There was disruption of the integrity of the myofibroblast monolayer, with formation of clumps (asterisks) surrounded by macrophages (arrowheads), 24 hrs after addition of surfactant (**B**, lower left image). Large myofibroblast clumps in the supernatant and some macrophages (different focal depth) still attached to the substrate were observed after 48 hrs (**B**, lower right), scale bar: 30 μm. Expression of smooth muscle α‐actin (red) and desmin (green) in myofibroblasts differentiated from fibroblasts (**C**), scale bar: 25 μm.

Using a coculture of myofibroblasts and macrophages, no changes were detected in myofibroblast cultures (Fig. 6B) or macrophages alone and if both were incubated individually with surfactant. These two cell types proved to be non‐reactive with one another if cocultured (Fig. 6A). However, if surfactant was added to the coculture medium, 24 hrs after incubation, disruption of the integrity of the myofibroblast monolayer was observed, and some of these non‐adherent cells, similar to myofibroblasts suspended in the supernatant, were predominantly found as cell clusters (Fig. 6B). In addition, some macrophages were found on the edges of those clusters, which were observed in the form of clumps surrounded by macrophages. After 48 hrs, large clumps were observed in the supernatant, and some macrophages were still attached to the substrate, along with very few myofibroblasts (Fig. 6D). Myofibroblasts were phenotypically identified by expression of smooth muscle actin (red) and desmin (green) (Fig. 6C).

## Discussion

It is well known that after a gastrointestinal surgery or any intra‐abdominal procedure, the formation of intra‐abdominal adhesions with different degrees of severity is almost inevitable because of the disruption of peritoneal integrity. In this study, a rat model of adhesion induced by gastroenterostomy was chosen based on pilot studies and a previous experimental report 15. In the model used herein, firm adhesions had been developed by the fourth post‐operative week, which was not observed in 100% of animals before this time point. Topical surfactant therapy reduced the occurrence of such adhesions; this reduction was associated with reduced TGF‐β expression and increased MMP‐9 activity and, consequently, a decrease in collagen fibre content.

We were able to find only one description of the use of surfactant (Poractant and Beractant) to prevent abdominal adhesions in the literature 9. Despite promising results, the authors limited their observations to a histological study with haematoxylin and eosin staining. We chose to study adhesion formation on the 28th day after surgery because, at this point, collagen deposition with adhesion formation is both relevant and stable.

Recent studies have shown that surfactant may be structurally homologous to C1‐q, a protein in the complement cascade 16. Furthermore, a previous study revealed that surfactant is a member of the collectin family, with an N‐terminal collagen‐like domain and a C‐terminal carbohydrate‐binding domain 17. On the basis of these studies, we can assume that, just as collectins form trimers, surfactant molecules can join together to form a large oligomer and to compete for binding sites on collagen present in the extracellular matrix during collagen synthesis by myofibroblasts. This process could prevent collagen deposition and, consequently, adhesion in the region of the anastomosis and on the peritoneal surface of the liver capsule or other organs, as occurred in this study in animals given surfactant after gastroenterostomy.

Corroborating a previous study 18, our work showed that surfactant (Beractant) was able to induce increased gelatinolytic and collagenolytic activity in enteric tissue. Furthermore, the zymography gel showed a predominance of MMP‐9 compared with other metalloproteinases. These results are consistent with the findings of other authors, who observed increased expression and activity of MMP‐9 in inflammatory cells after treatment with surfactant 19. In addition to this effect of competition for collagen‐binding sites, surfactant appears to increase MMP‐9 activity, leading to the well‐known effect of degradation of denatured collagen (gelatin) and type IV collagen 19. In fact, our *in vitro* results showed that surfactant disrupts the adhesion of myofibroblasts to the substrate if they are cocultured with macrophages. This same effect was not observed in myofibroblast and macrophage monocultures whether in the presence or absence of surfactant.

Our molecular biology analysis has shown that treatment of rats with surfactant after the induction of intra‐abdominal adhesions reduced synthesis of mRNA for the profibrotic protein TGF‐β. As TGF‐β induces fibroblasts to excessively produce, deposit and contract extracellular matrix, this cytokine plays a pivotal role in the fibrotic response and, therefore, in the formation of adhesions. Accordingly, we can assume that, in our model, surfactant treatment had a reducing effect on TGB‐β‐induced fibrosis.

As observed with TGF‐β, our analysis of mRNA levels for type III procollagen showed that surfactant administration after the induction of adhesions reduced synthesis of PCIII. Type III procollagen can be derived from the synthesis of new type III collagen or from the degradation of existing type III collagen fibrils. Therefore, the presence of type III procollagen can be considered as an indicator of fibrogenesis and intense inflammatory activity in which collagen breakdown occurs 20. In this context, our data suggest a possible anti‐fibrogenic effect of surfactant through a reduction in new type III collagen synthesis.

Because aberrant apoptosis has been frequently associated with worsening stages of fibrosis 21, we decided to test the effects of surfactant on the induction of apoptosis *via* caspase‐3 activation. Our analysis of pro‐caspase 3 mRNA levels and of activated caspase‐3 protein expression by immunoblotting showed that surfactant can play a critical role in the prevention of apoptosis. Based on these findings, we can assume that this anti‐apoptotic effect of surfactant may have been responsible for the inhibition of adhesion formation in the region of the anastomosis. Recent evidence has suggested that the Th1/Th2 immune balance may play important roles in fibrogenesis 22. The fibrogenic response has been associated with drastically lower expression of interferon‐γ and consequent failure of Th1 polarization 22. Therefore, the anti‐apoptotic effect of surfactant could promote Th1 polarization, and the subsequent Th1 immune response would play a role in reducing intestinal collagen deposition. However, further studies will be necessary to elucidate this mechanism.

The molecular cause of fibroblast activation is still unknown. Some authors have reported a direct correlation between KGF produced by fibroblasts and induction of fibrogenesis in skin 23. However, other studies have shown that KGF secreted by fibroblasts prevents pulmonary fibrosis 24. In our particular case, we observed that treatment with surfactant reduced KGF mRNA levels, and this reduction was associated with prevention of adhesions.

Isolectin B4 binds to glycoconjugates containing terminal alpha‐D‐galactose present on the surface of monocytes and macrophages, and has been reported to be useful for discriminating between several subtypes of macrophages in the rat bowel 25. Although no study has evaluated IB4 activity after a gastrointestinal anastomosis, we observed that IB4+ cells, which are consistent with infiltrating macrophages, were frequently present in the region of the gastrointestinal anastomosis in both tested groups. Although some authors claim that alpha‐D‐galactose epitopes on macrophage surfaces accelerate recruitment and promote wound repair 26, we were unable to verify this effect in our model, as there was no significant difference in the number of IB4+ cells in the GE group after treatment with surfactant. Therefore, additional studies are needed to determine the effect of surfactant on the recruitment and activation of macrophages in gastrointestinal anastomotic regions.

Taken together, our data suggest that the downregulation of TGF‐β, KGF and procollagen III, as well as the upregulation of expression and surfactant‐mediated activity of MMP‐9 in the gastroenterostomy region, contribute to the disruption of fibrogenesis by regulation of extracellular matrix deposition and degradation, respectively. Moreover, surfactant may also mediate adhesion‐reducing effects by increasing the proteolytic activity of MMP‐9 in activated ED1‐positive macrophages. ED1 has been widely used as a marker for rat macrophages and is found predominantly on the lysosomal membrane. Therefore, ED1 expression has been closely related to the phagocytic activity of macrophages, and it has been detected in inflammatory bowel diseases such as experimental granulomatous colitis 27, 28. This study showed that an increased number of ED1‐positive macrophages in a rat model of experimental colitis induced by peptidoglycan–polysaccharide was not significantly attenuated by blocking monoclonal antibodies for an immunoglobulin superfamily adhesion molecule, suggesting that other mechanisms can play important roles in macrophage infiltration 27. A previous study revealed that MMP‐9 expression in corneal tissue is higher in ED1‐positive macrophages in a possible mechanism mediated by autocrine loops with positive feedback 29. Similarly, this mechanism could have occurred in our model, because these high levels of MMP‐9 are responsible for an increased number of ED1‐positive macrophages (activated macrophages), which are potentially able to reduce fibrogenesis. Thus, our objective was to determine whether macrophage subpopulations expressed MMP‐9 and may participate in collagen degradation. Concerning macrophage analysis, surfactant therapy resulted in no effect, since no differences were observed between GE and GE‐S animals after counting IB4‐positive cells expressing MMP‐9. However, the number of activated macrophages immunostained with an anti‐ED1 antibody was significantly higher in GE‐S compared to GE group.

A previous study revealed that latent gelatinase B, as well an active form, was significantly increased in culture medium (24 hrs) of activated alveolar macrophages from bronchoalveolar lavage of patients with idiopathic pulmonary fibrosis 30. Thus, it is reasonable to hypothesize that surfactant induced a macrophage‐mediated gelatinolytic activity in our coculture model with myofibroblasts since the addition of surfactant caused disruption of the integrity of the myofibroblast monolayer.

**Figure 7:**
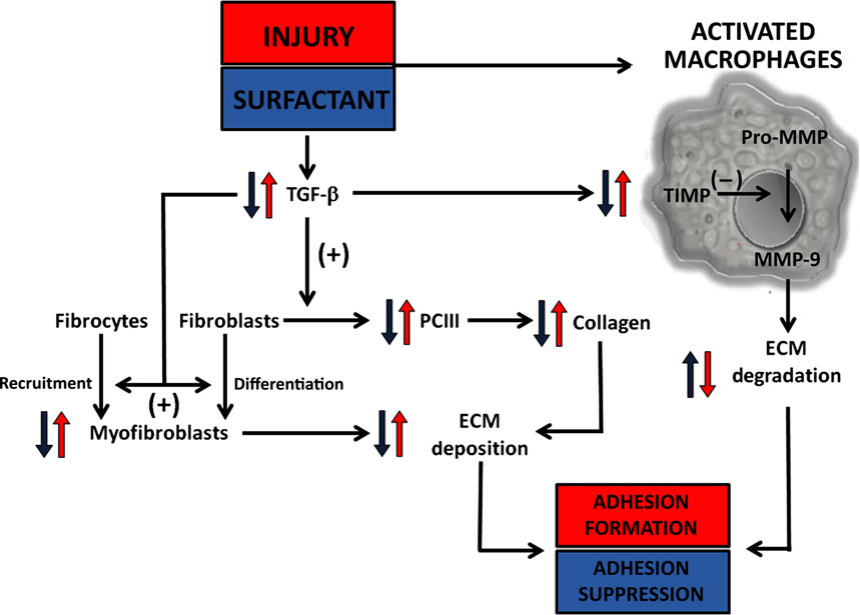
Schematic representation of peritoneal adhesions and the putative effects of treatment with pulmonary surfactant. Pulmonary surfactant reduces the production of TGF‐β, inhibiting the differentiation of fibrocytes to myofibroblasts and the production of PCIII. Furthermore, surfactant may have a direct effect on macrophage activation, inducing extracellular matrix degradation and resolution of fibrosis *via* MMP‐9 production. Blue arrows denote the effects of surfactant and the mechanisms of adhesion suppression. Red arrows show the effect of the peritoneal injury and adhesion formation.

Based on our results and on the existing literature 31, we have proposed the putative mechanisms involved in the surfactant‐mediated suppression of adhesion. We hypothesize that surfactant may act in the early stages of fibrogenesis, downregulating TGF‐β signalling and thereby altering myofibroblast differentiation and, consequently, ECM deposition. Simultaneously, surfactant may act on the expression and activity of MMP‐9, leading to an increase in ECM degradation (Fig. 7).

Surfactant is easy to apply over the surgical field and does not require room temperature control or any type of previous preparation. It has excellent biocompatibility and has long been used for the treatment of neonatal respiratory distress syndrome. However, some limitations of this study must be taken into account. Although the composition of rat peritoneal surfactant does not differ substantially from that of humans 32, further studies with other species of animals, such as pigs and primates, will be necessary. Unlike in the rat, a large volume of surfactant will be required to cover the surface area of the operative field in human patients undergoing abdominal surgery, and the high cost of such administration should be considered. Finally, the possibility of adverse effects or complications of surfactant therapy that will require close follow‐up should be considered in future dose‐dependent studies.

In conclusion, our results showed that topical administration of surfactant effectively prevented intra‐abdominal adhesions in a rodent model. Such therapeutic management has the potential to reduce the high social and financial cost required to deal with recurrent surgical interventions caused by postoperative adhesions. Further studies will be necessary to establish the applicability of this drug in human surgery.

## Conflicts of interest

The authors confirm that there are no conflicts of interest.
